# The vicinal difluoro motif: The synthesis and conformation of *erythro*- and *threo*- diastereoisomers of 1,2-difluorodiphenylethanes, 2,3-difluorosuccinic acids and their derivatives

**DOI:** 10.1186/1860-5397-2-19

**Published:** 2006-10-02

**Authors:** David O'Hagan, Henry S Rzepa, Martin Schüler, Alexandra M Z Slawin

**Affiliations:** 1 School of Chemistry, University of St Andrews, Centre for Biomolecular Sciences, North Haugh, St Andrews, Fife, KY16 9ST, UK; 2 Department of Chemistry, Imperial College of Science Technology and Medicine, Imperial College London, SW7 2AZ, UK

## Abstract

**Background:**

It is well established that vicinal fluorines (RCHF-CHFR) prefer to adopt a *gauche* rather than an *anti* conformation when placed along aliphatic chains. This has been particularly recognised for 1,2-difluoroethane and extends to 2,3-difluorobutane and longer alkyl chains. It follows in these latter cases that if *erythro* and *threo* vicinal difluorinated stereoisomers are compared, they will adopt different overall conformations if the fluorines prefer to be *gauche* in each case. This concept is explored in this paper with *erythro*- and *threo*- diastereoisomers of 2,3-difluorosuccinates.

**Results:**

A synthetic route to 2,3-difluorosuccinates has been developed through *erythro*- and *threo*- 1,2-difluoro-1,2-diphenylethane which involved the oxidation of the aryl rings to generate the corresponding 2,3-difluorosuccinic acids. Ester and amide derivatives of the *erythro*- and *threo*- 2,3-difluorosuccinic acids were then prepared. The solid and solution state conformation of these compounds was assessed by X-ray crystallography and NMR. *Ab initio* calculations were also carried out to model the conformation of *erythro*- and *threo*- 1,2-difluoro-1,2-diphenylethane as these differed from the 2,3-difluorosuccinates.

**Conclusion:**

In general the overall chain conformations of the 2,3-difluorosuccinates diastereoisomers were found to be influenced by the fluorine *gauche* effect. The study highlights the prospects of utilising the vicinal difluorine motif (RCHF-CHFR) as a tool for influencing the conformation of performance organic molecules and particularly tuning conformation by selecting specific diastereoisomers (*erythro* or *threo*).

## Background

**Scheme 1:**
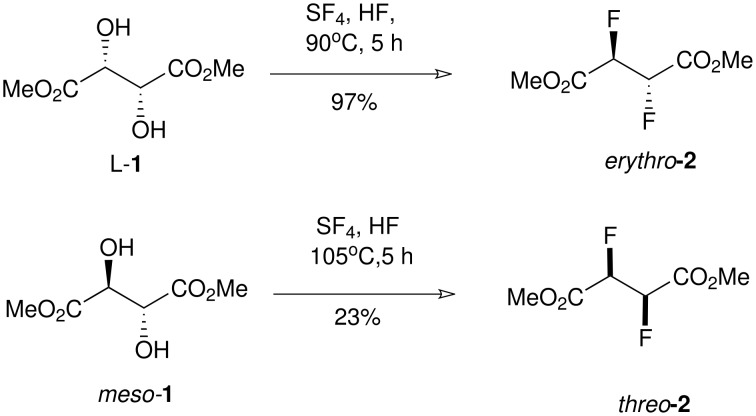
Synthesis of *vicinal* dimethyl difluorosuccinates. The conversion of the tartrates **1** with SF_4_ and HF [6–7].

**Scheme 2:**
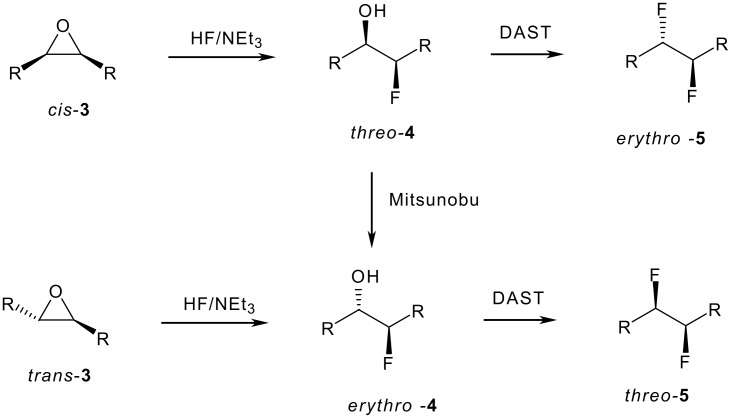
Schlosser's route to vicinal *erythro*- or *threo*- difluoro alkanes **5** [13].

Of the 298,876 registered fluorinated structures in the Beilstein Chemical Database (for 2005) only 279 compounds contain a genuine *vicinal* difluoro motif-CHF-CHF- and only 12 crystal structures of this motif are deposited in the Cambridge Structure Data Base. The relatively rare presence of this motif may partly be attributed to the difficulty of their selective synthesis. It remains a synthetic challenge to prepare vicinal difluorocompounds efficiently and particularly in a stereoselective manner. There are attractive reasons to explore this motif. It is well known that the conformation of 1,2-difluoroethane is influenced by the fluorine *gauche effect*, where the fluorines prefer to be *gauche* rather than *anti* to each other [1]. This preference extends to 2,3-difluorobutane [2], and we have shown that *erythro*- and *threo*-9,10 difluorostearic acids have very different physical properties [3], the origin of which appears to lie in the different conformational preferences associated with the vicinal difluoro- motif for each diastereoisomer. Early synthetic methods to vicinal difluoro compounds have involved direct fluorination of alkenes with for eg. elemental fluorine (F_2_) [4] or XeF_2_ [5]. Such methods however are either difficult to carry out in a standard laboratory environment or they suffer from very poor stereoselectivity. The direct conversion of vicinal diols to vicinal difluorides has been explored with some success. For example both *erythro* and *threo* stereoisomers of dimethyl 2,3-difluorosuccinic acid were obtained either from methyl esters of the L-tartrate **1** or the *meso*-tartrate **1** by treatment with SF_4_/HF (Scheme 1) [6–7]. Conversion to the product *erythro*-**2** proved efficient (97%) but that to *threo*-**2** was poor (23%) due to competing elimination. The preparation of the *erythro* isomer of **2** is attractive on a large scale although SF_4_ has to be used with care and it is not amenable to reactions on a small scale. Our attempts to replace SF_4_ with DAST failed in trying to develop an analogous small scale laboratory process. Deoxofluor is finding use in the stereoselective conversion of vicinal diols to vicinal difluorocompounds and seems less prone to elimination than DAST [8]. In addition, Deoxofluor has been reported to be thermally more stable than related aminosulfur trifluoride reagents which allows the conversions to be carried out safely at elevated temperatures [9–10]. The stereoselective conversion of vicinal ditriflates to vicinal difluorides by treatment with TBAF has also been reported, particularly for the synthesis of 3,4-difluoropyrrolidine ring systems, and these reactions are finding currency in pharmaceutical products [11–12]. Schlosser *et al*. [13] have developed the most practical and straightforward method to access a variety of *erythro*- or *threo*- vicinal difluoro compounds in a diastereoselective manner, using either c*is*- or *trans*- epoxides **3** obtained directly from either the *Z*- or the *E*- alkenes. (Scheme 2). Ring opening of the epoxides **3** with HF-amine reagents generate the corresponding *threo*- and *erythro*- fluorohydrins **4** in largely a stereoselective manner. The resulting fluorohydrins **4** can then be converted to the *erythro*- or the *threo*- vicinal difluoro compounds **5** with reagents such as DAST [9–10] or Deoxofluor [8,14], although elimination products often compete with fluoride substitution depending on the nature of the substrate.

**Scheme 3:**
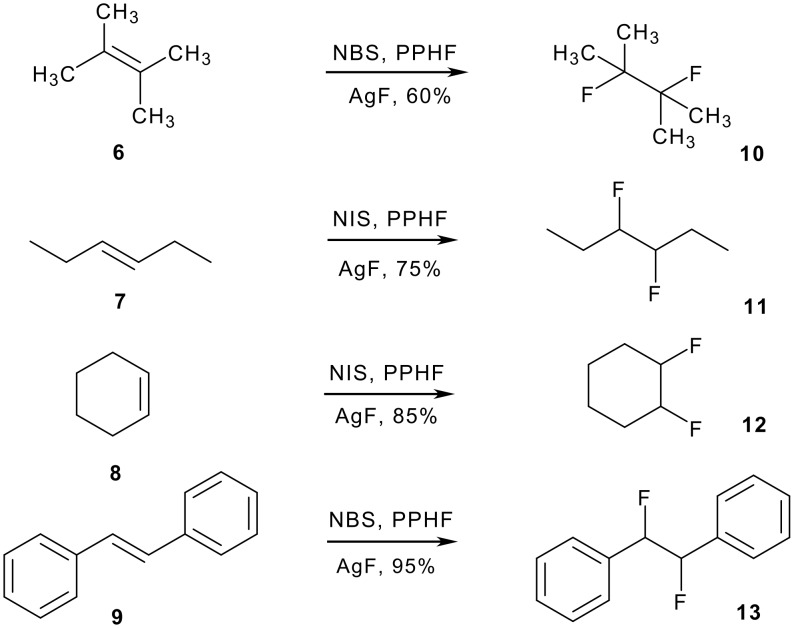
Halofluorination of electron-rich alkenes with *in situ* fluoride displacement generates *vicinal* difluoro products. PPHF is Olah's reagent, pyridinium poly(hydrogen fluoride) [15].

V*icinal* difluoro compounds have been prepared by halo(bromo/iodo)fluorination of alkenes followed by halide substitution with silver fluoride [15]. The reaction has been applied to a variety of alkenes some of which (eg **6**-**9**) are illustrated in Scheme 3.

We were interested in accessing diastereomerically pure samples of *erythro*- and *threo*- 2,3-difluorosuccinic acids **19**. The preparation of stereoisomers of 2,3-difluorosuccinic acids, has involved conversions of tartaric acids (esters) [6–7], as described above in Scheme 1. Other approaches have involved the direct fluorination of fumaric acid [16] and the catalytic hydrogenation of 2,3-difluoromaleic acid [7], but these processes result in significant by-product formation and gave only poor yields of the desired products. Our alternative approach chose to explore the oxidation of the aromatic rings of *erythro*- and *threo*- diastereoisomers of 1,2-diphenyl-1,2-difluoroethane **13**, exploiting the ability of the phenyl group to act as a latent carboxylic acid [17]. This paper describes these studies and we report the solid and solution state conformation of the *erythro*- and *threo*- diastereoisomers of **13** and the resultant 2,3-difluorosuccinic acid stereoisomers and some of their derivatives. Some of these results have recently been communicated [18]. The study suggests that the vicinal fluorine *gauche* effect can have a significant influence on the conformation of the 1,2-difluorosuccinates.

## Results and Discussion

### Synthesis of erythro- and threo- 1,2-diphenyl-1,2-difluoroethanes 13

**Scheme 4:**
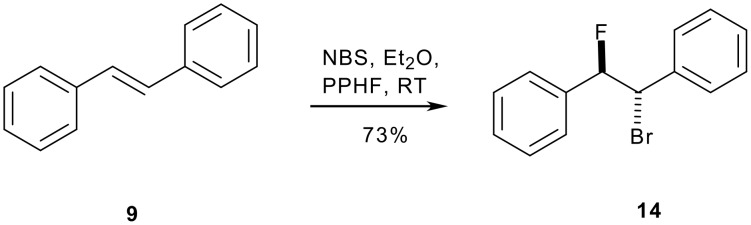
Bromofluorination of stilbene [19].

Stilbene **9** is readily converted to its bromofluoro adduct by treatment with NBS and pyridine:HF following Olah's method [19] (Scheme 4).

**Scheme 5:**
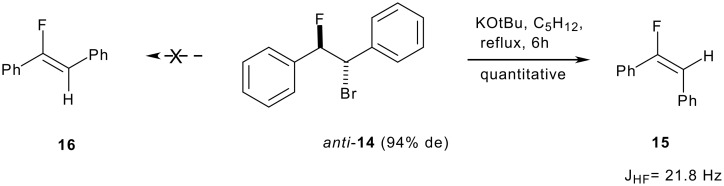
Treatment of *anti*-**14** with base generated the *E*-fluorostilbene **15** by an *anti* elimination mechanism.

In our hands product **14** was generated with a diastereoselectivity of 94%. The predominant *anti* stereochemistry of **14** was established from the coupling constants of the olefin products obtained after a dehydrobromination reaction. The elimination of hydrogen bromide from such β-fluorobromides had been explored previously, and the reaction proceeds in a stereospecific manner to generate either *E* or *Z* fluoroalkene products [20]. Accordingly treatment of **14** with potassium *tert*-butoxide in a refluxing solution of hexane or pentane lead to the exclusive formation of the *E*-alkene **15** as judged by the ^3^*J*_HF_ coupling constant of 21.1 Hz obtained from ^19^F-NMR. This is indicative of a stereospecific *anti*-elimination of hydrogen bromide from **14** to generate **15** with a cisoid relationship between H and F, rather than compound **16** which would have a *trans* relationship and a much larger ^3^*J*_HF_ coupling constant (~30 Hz), and reinforces the stereochemical assignment made to **14** as illustrated in Scheme 5 [21].

**Scheme 6:**
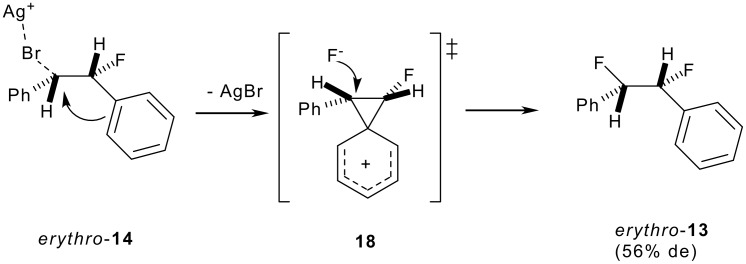
Hypothesis for the predominent retention of configuration during fluoride substitution *via* phenonium intermediate **18**.

Substitution of the bromine in *anti*-**14** with fluorine was accomplished by treatment with Ag(I)F in acetonitrile in the dark. Under these conditions, the substitution proceeds smoothly to *erythro*-**13** but only in 56% de indicating a significant loss of stereochemical control during the reaction. The predominant stereochemical outcome of the fluorine substitution reaction suggests a double inversion mechanism as the major *erythro*-**13** isomer must arise by replacement of the bromine of *anti*-**14** by fluorine with an overall retention of configuration. Various examples of anchimeric assistance by phenyl groups have been reported [22] and in this case a carbocation is most reasonably generated which finds benzylic as well as anchimeric stabilisation *via* phenonium ring formation **18** with the β-phenyl group as illustrated in Scheme 6.

**Scheme 7:**
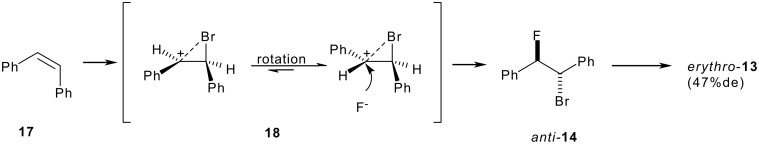
Proposed C-C bond rotation during the preparation of **14** from *cis*-stilbene.

Isolation of the minor *threo*-**13** isomer required careful chromatography. In order to improve the synthesis of *threo*-**13** a reaction with *cis*-stilbene **17** was investigated. The one pot process with NBS, PPHF and Ag(I)F again proceeded smoothly however it also gave *erythro*-**13** as the major product of the reaction, although with a reduced diastereoisomeric ratio (47% de) more suitable for *threo*- **13** isolation. The bias towards *erythro*- **13** in this case is clearly a result of internal rotation about the central carbon-carbon bond, to relieve a steric clash between the vicinal phenyl groups, after initial formation of an intermediate bromonium ion **18** as illustrated in Scheme 7.

**Figure 1:**
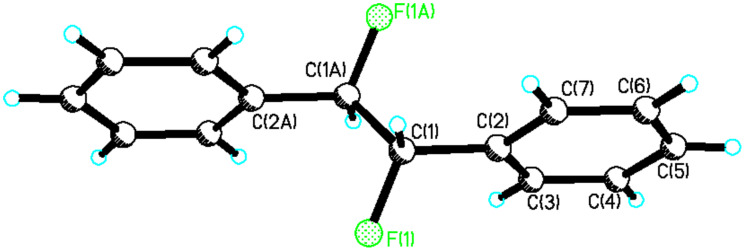
Crystal structure of *erythro*-**13**.

*Erythro* **13** was readily purified after several crystallisations whereas isolation of the *threo* isomer of **13** was more challenging. Partial separation of *threo*-**13** was achieved by means of preparative thin layer chromatography. The enriched diasteroisomeric mixture could be crystallised to purity and crystals suitable for X-ray structure analysis were obtained (Figure 1). In the solid state *erythro*-**13** adopts a conformation in which the phenyl substituents are *anti* to each other, with a Ph-C-C-Ph torsion angle of 180°. As a result the C-F bonds also align *anti* with respect to each other with a F-C-C-F torsion angle also close to 180°.

**Figure 2:**
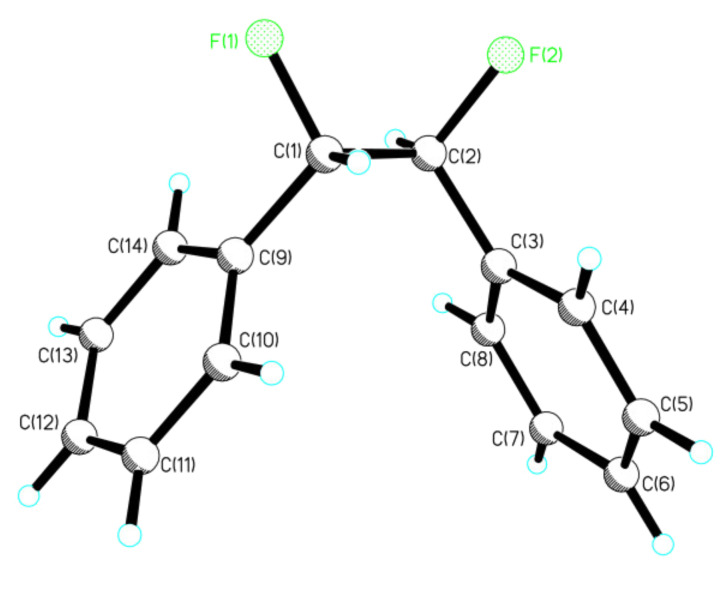
X-ray structure of *threo*-**13**.

A stereochemical mixture enriched in favour of *threo*-**13** was crystallised to purity and a suitable crystal was used for X-ray structure analysis. The resultant structure is shown in Figure 2.

The most obvious feature of this structure is the perhaps unexpected *gauche* relationship between the phenyl rings which places the fluorine atoms also *gauche* to each other. This superficially suggests that the fluorine *gauche* effect is over-riding any steric repulsion between the phenyl rings. To explore the significance of these solid state conformations further, NMR solution studies and *ab initio* analysis, exploring the preferred conformations for each of the diastereoisomers was carried out.

### NMR studies on *erythro*- and *threo*-13

**Figure 3:**
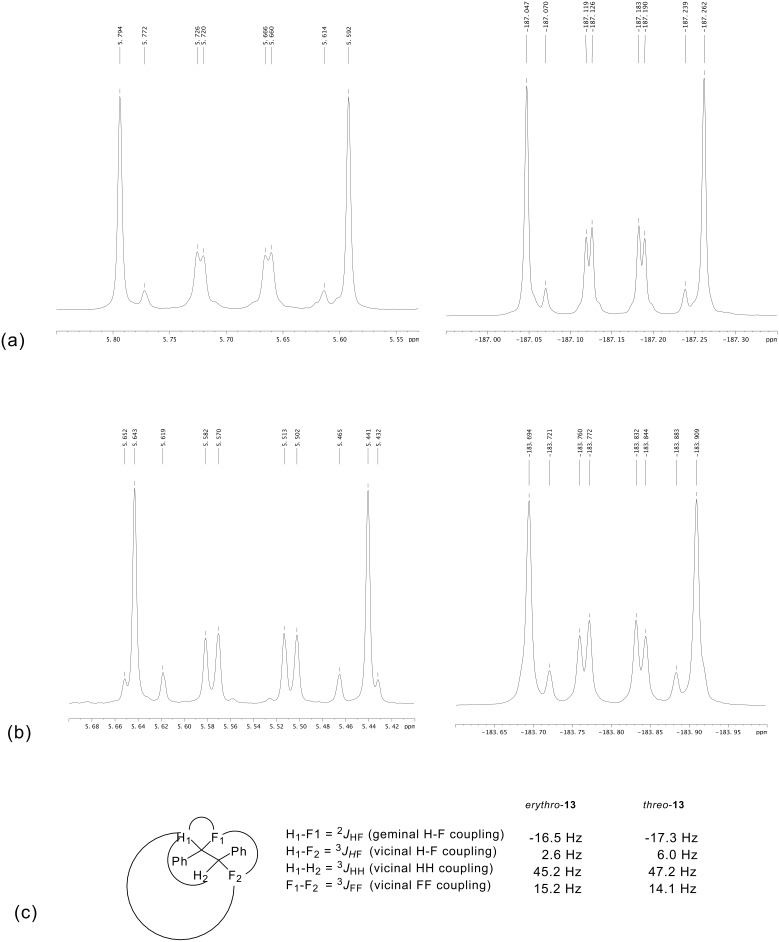
Expanded regions of the second order AA'XX' spin systems in the ^1^H-NMR (left) and ^19^F-NMR spectra (right) of *erythro*-**13 (a)**, *threo*-**13 (b)** and the four individual coupling constants for the central ^1^H and ^19^F nuclei are given in **(c)**.

The most obvious feature in the ^1^H- and ^19^F- NMR spectra of the diastereoisomers of **13** is the coupling pattern from the AA'XX' spin system (Figure 3). Due to the chemical equivalence but magnetic non-equivalence of the F and H atoms a second-order spectrum is generated in each case.

**Figure 4:**
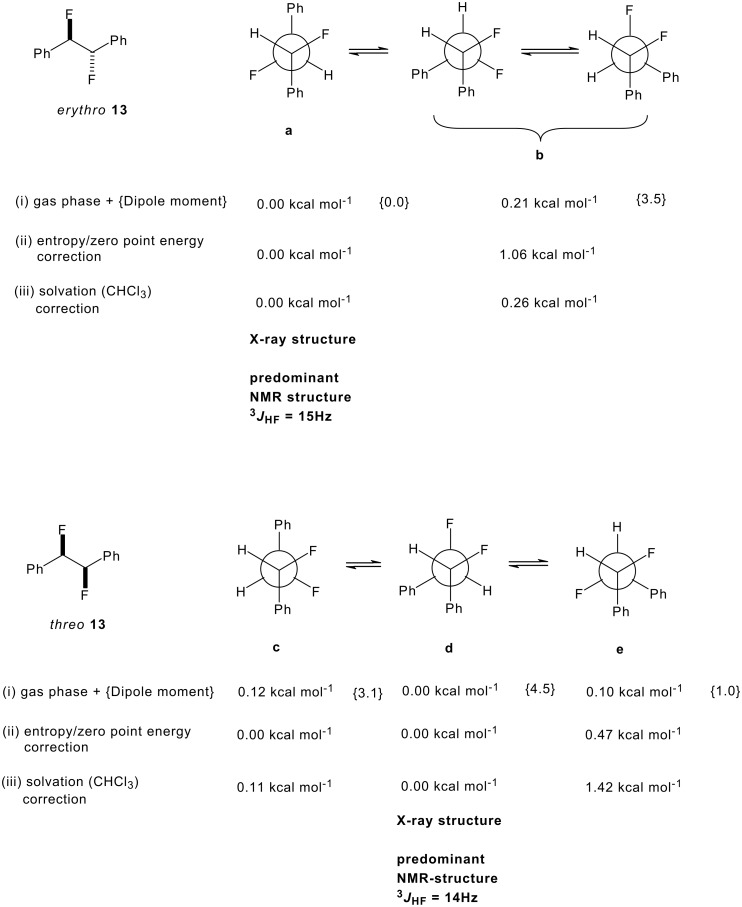
NMR coupling constants and calculated relative energies (kcalmol^-1^) of the staggered conformers of *erythro*- and *threo*- **13** calculated at the B3LYP//cc-pVTZ level. Relative energies (i) in the gas phase (ii) corrected for entropy and zero point energy differences and (iii) using a solvation model are reported. Calculated dipole moments {μ} are also given.

Measuring of coupling constants from such second-order spectra has been described by Abrahams *et. al*. [23] although the analysis requires an intuitive fitting of values to specific coupling relationships. These deduced values are tabulated in Figure 3c. The large values of 45.2 & 47.2 Hz clearly correlate to the *geminal* ^2^*J*_HF_ coupling, and the values of 15.2 & 14.1 Hz to the *vicinal* ^3^*J*_HF_ coupling. The smaller coupling constant of 2.6 & 6.0 Hz most appropriately correlate to the ^3^*J*_HH_ couplings, and thus, the value of -16.5 & -17.3 Hz is assigned to the *vicinal* ^3^*J*_FF_ coupling. The ^19^F NMR spectrum can similarly be assigned in each case and reinforced these values. The magnitude of the different vicinal NMR coupling constants can be rationalised in terms of rotational isomerism of the individual diastereoisomers. Only the three staggered conformations for *erythro*- and *threo*- **13** are considered (Figure 4).

It is not obvious from the NMR data which of **a** or **b** is the favoured solution conformation for the *erythro* isomer. We infer a significant contribution from rotamer **b** where the C-H bonds are *gauche* on the basis of the small ^3^*J*_HH_ value (2.6 Hz), however the relatively small ^3^*J*_HF_ value (15 Hz) suggests two C-H and C-F *gauche* relationships implying a contribution from rotamer **a**. Rotamer **a** most closely resembles the X-ray structure for *erythro*-**13** shown in Figure 1. The situation is much clearer for *threo*-**13**. The relatively large ^3^*J*_HH_ coupling constant (6.0 Hz) and the small ^3^*J*_HF_ coupling constant (14 Hz) suggests a significant population of rotatmer **d**. This isomer has the vicinal C-H bonds *anti* to each other and both of the C-F/C-H and C-F/C-F bonds *gauche*. This is also the preferred conformation for this compound in the solid state (X-ray structure in Figure 2).

### Conformational energy calculations on *erythro* and *threo*-13

Due to the ambiguous solution state study particularly for *erythro*-**13**, *ab initio* calculations were carried out at the B3LYP//cc-pVTZ level exploring absolute energies of the three staggered conformers of both *erythro*- and *threo*- **13** [24–25]. The geometries were optimized at this level for a gas phase model, and corrected for entropy and zero-point energy differences at this level. A separate solvation correction (chloroform) was applied using a continuum model (PCPM) and the larger cc-pV5Z basis set (using pVTZ geometries). Chloroform was studied in an attempt to relate the calculated values to the NMR solution conformations (*vide infra*). The relative energy data and dipole moments for each diastereoisomer are presented in Figure 4. The calculated conformations and energies can be viewed at http://www.ch.ic.ac.uk/rzepa/ohagan/ (see Supporting Information File 1).

Of the three staggered conformers of the *erythro*-**13** isomer two are enantiomeric and have identical energies thus analysis of *erythro*-**13** is reduced to a comparison of the energies of conformers **a** and **b**. Conformer **a** emerges as the more stable in the gas phase, with this stability originating predominantly from entropy and zero-point energy corrections (1.06 kcal/mol). This is also the conformer that most closely represents the X-ray structure (Figure 1). The solvent correction (which takes into account free energy differences associated with the solvent cavity, but does not allow for free energy differences arising from vibrational terms) does not alter the relative energies of **a** and **b,** despite **a** having a zero dipole moment and **b** having a relatively large value (3.5D) [26]. Although the more polar **b** should perhaps gain more from electrostatic solvation, it has a smaller solvent accessible surface area (239A^2^ vs 246 A^2^ for **a)** and these two appear to cancel in their overall effect on the relative energies. Our best estimate of the relative stability of **a** and **b** is about 1.0 kcal/mol in favour of the former as noted above. Thus structure **a** does not conform to a fluorine *gauche* effect and appears to be dominated by solvation of the *trans* relationship of the aryl rings and the zero dipole moment, although the smaller ^3^*J*_HH_ coupling of 2.6 Hz and the slightly larger ^3^*J*_HF_ coupling of 15 Hz in the NMR, measurement does suggest some contribution of conformer **b** in solution.

The *threo*-**13** isomer has three distinct staggered conformations; **c**, **d** and **e**. Computationally, this requires modelling the subtle balance between the correlation effects due to *gauche* fluorine atoms and those due to *gauche* phenyl rings. In the gas phase (entropy and zero energy corrected) conformers **c** and **d** are iso-energetic. The dipole moments for these conformers vary significantly, with **d** > **c** > **e**. As with the *erythro* isomer, the greater solvation for **d** is partially offset by a smaller solvent-accessible surface (238 vs 247 Å ^2^ for **c**). Although **d** is slightly favoured in this model (by 0.11 kcal/mol), this is significantly smaller than the NMR estimate and may reflect a limitation of the solvation model. Taking all of the data together (theory, X-ray and NMR) conformer **d** appears to be the most favoured conformer for *threo*-**13** with both the fluorine and the phenyl rings *gauche*, despite its larger dipole moment.

### 2,3-Difluorosuccinic acids 19

**Scheme 8:**
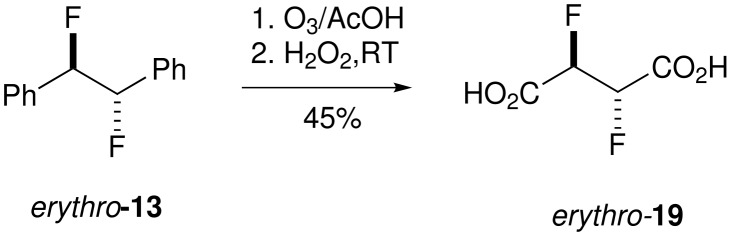
Synthesis of *erythro*-**19** *via* ozonolysis of *erythro*-**13**.

**Figure 5:**
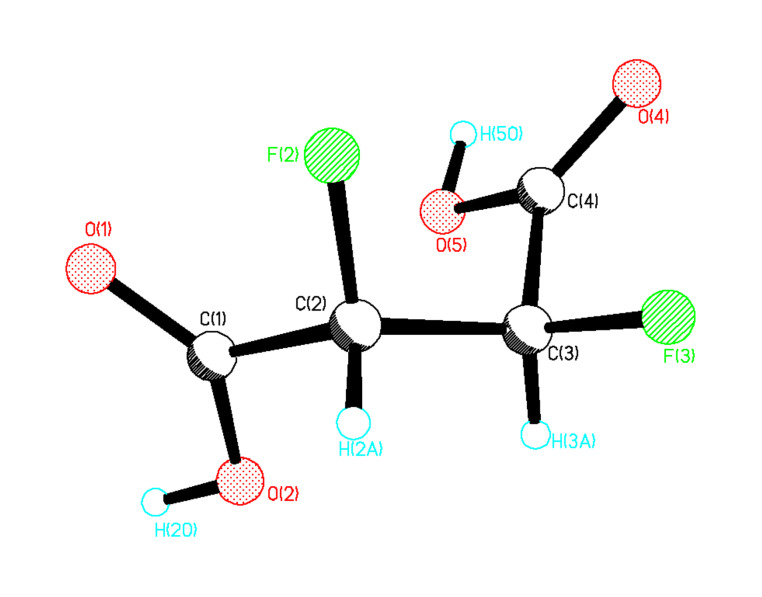
X-ray structure of *erythro*-**19**.

The synthesis of the 2,3-difluorosuccinic acid diastereoisomers **19** was explored by the oxidation of the aryl rings of **13** to carboxylic acids. Oxidative degradation of aromatic rings has been achieved by RuCl_3_/NaIO_4_ oxidation [17] however this method proved unsatisfactory in our hands and lead to poor conversions and a complex product mixture. As an alternative strategy ozonolysis in acetic acid, with a hydrogen peroxide work-up was explored [27–28], and this proved successful as illustrated in Scheme 8. For example, reaction of a 4:1 mixture of *erythro*- and *threo*- **13** led to the formation of **19** also in a 4:1 ratio of diastereoisomers. *Erythro* 2,3-difluorosuccinic acid **19** was obtained in a modest yield as a single stereoisomer from a stereochemically pure sample of the *erythro* **13**. A crystal of *erythro*-**19** suitable for X-ray analysis was obtained after sublimation, and the resultant structure is shown in Figure 5.

**Figure 6:**
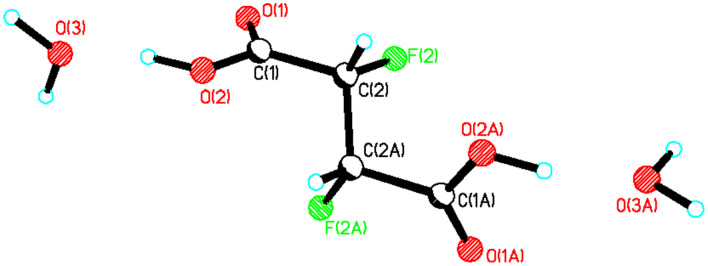
X-ray structure of *threo*-**19**.

In the X-ray crystal structure of *erythro*-**19** both of the carboxylic acid carbonyl oxygens adopt a *syn* periplanar conformation with respect to the C-F bonds. In the crystal packing, the carboxylate groups of two neighbouring molecules are hydrogen bonded and this clearly determines the three dimensional structure of the unit cell. The *threo*-**19** diastereoisomer was prepared by a similar aryl oxidation reaction on a diastereomerically pure sample of *threo*-**13** and this allowed crystallisation of a sample of racemic *threo*-**19**. The X-ray structure in Figure 6 shows the molecule in an extended chain conformation with both of the C-F bonds *gauche* to each other. One molecule of water is bound for every succinic acid molecule and this water clearly participates in hydrogen bonding to the carboxylic acid groups.

**Scheme 9:**
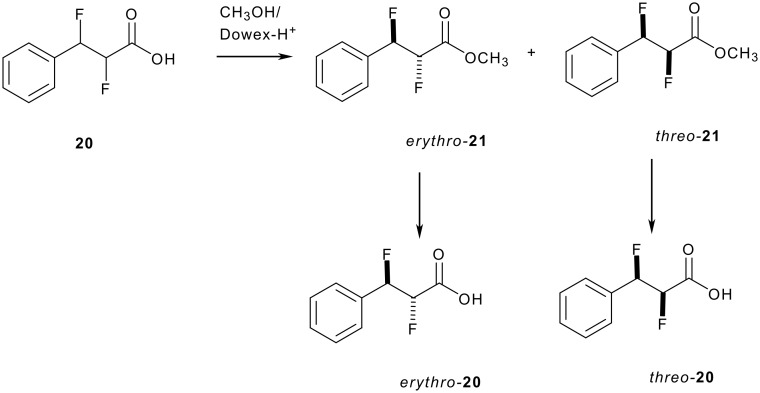
Strategy for the preparation of diastereoisomers of *erythro*- and *threo*- **20**.

The major by-product of the ozonolysis reaction of **13** was the *vicinal* difluorophenylpropionic acid **20** as a mixture of stereoisomers. The compound was purified by esterification with methanol to generate esters **21**. These diastereoisomers could be separated by chromatography and then hydrolysis was achieved under acidic conditions, followed by recrystallisation as illustrated in Scheme 9 to generate racemic, but diastereomerically pure samples of *erythro*- and *threo*- **20**.

**Figure 7:**
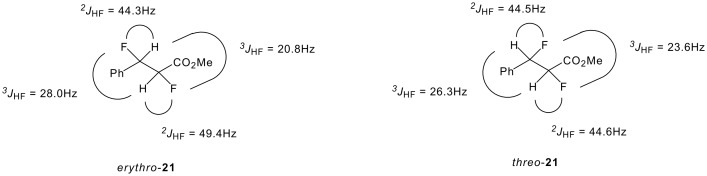
NMR (CDCl_3_, RT) coupling constants of *erythro*- and *threo*- 2,3-difluoro-3-phenylpropionates **21**.

The ^3^J_HH_ coupling constants of esters **21** remain small (2.8-3.6 Hz) and indicate a *gauche* relationship between these vicinal hydrogens as summarised in Figure 7. It follows that in each case the fluorines will be predominantly *gauche* to each other.

**Figure 8:**
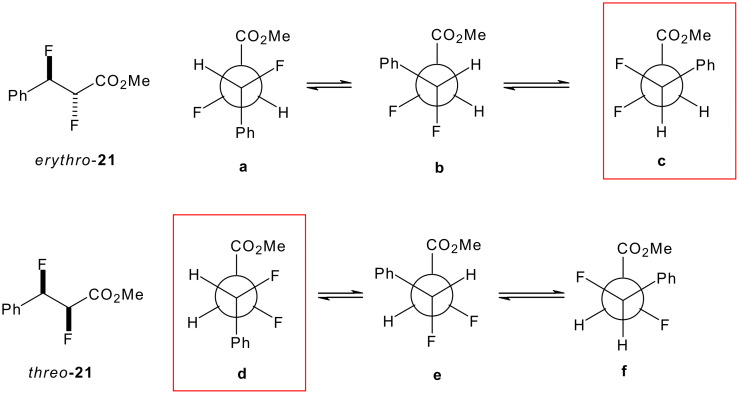
Newman projections showing the staggered conformations of *erythro*- and *threo*- **21**.

The observed values for *erythro*- **21** report a maximal ^3^*J*_HF_ coupling constant for the β-fluorine (28 Hz), but an intermediate one for the α-fluorine (20.8 Hz). This suggests a conformational preference for rotamer **c,** which has a *gauche* vicinal fluorine relationship, over **a** (Figure 8). For the *threo*- **21** isomer, there are two *vicinal* ^3^*J*_HF_ couplings of similar and large magnitude (26.3 and 23.6 Hz) suggesting that rotamer **d**, with two *trans* ^3^*J*_HF_ relationships and again with the fluorines *gauche*, is the most significant contributor to the solution conformation.

**Figure 9:**
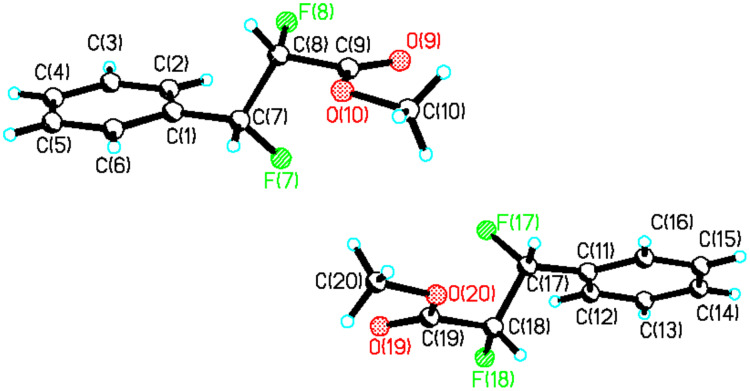
X-ray structure of methyl *threo*- **21**.

In the solid state structure of *threo*-**21** in Figure 9, the C-F bonds adopt a *gauche* relationship and the phenyl and ester groups are *anti* to each other. This is consistent with the preference for rotamer **d** found in solution. Attempts to crystallise *erythro*-**21** as its free carboxylic acid resulted only in the formation of amorphous material and thus a comparison of solution and solid state structures was not possible for this isomer.

### Amides of 2,3-difluorosuccinic acid

**Figure 10:**
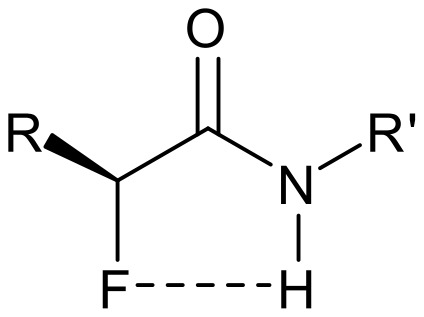
The preferred conformation of α-fluoroamides has the C-F and amide carbonyl *anti-planar* [29–30].

It was an objective of this research to explore the conformational preferences of amides of 2,3-difluorosuccinamides, particularly as we have previously noticed a conformational presence in α-fluoroamides [29], where the C-F bond aligns *anti* and planar to the amide carbonyl as illustrated in Figure 10. This adds an additional conformational constraint to these amides with a barrier to rotation around the C(CO)-C(F) bond of around 7-8 kcal mol^-1^. The preference of the C-F bond in α-fluoroamides to align *anti periplanar* to the carbonyl bond can be rationalized in terms of C-F bond and amide dipole relaxation as well as N-H...F hydrogen bonding [30].

**Scheme 10:**
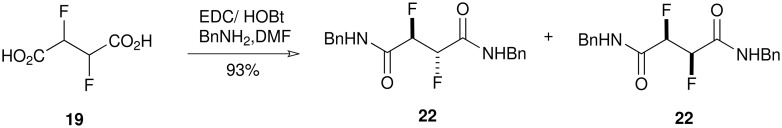
The synthesis of stereoisomers of *erythro*- and *threo*- **22**. These isomers could be separated by chromatography.

The solution and solid state structures of 2,3-difluorosuccinate benzylamides **22** have been evaluated. These compounds were prepared by a straightforward EDCI amide coupling between benzylamine and 2,3-difluorosuccinate **19** as shown in Scheme 10.

**Figure 11:**
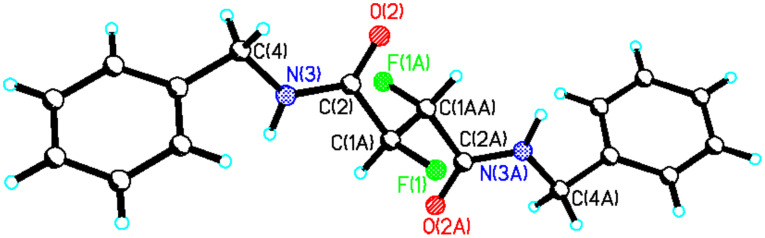
X-ray structure of *erythro*-**22**.

The diasteroisomers of **22** were separated by silica gel chromatography and recrystallisation of each allowed their X-ray structures to be compared. The structure of *erythro*-**22** is illustrated in Figure 11.

**Figure 12:**
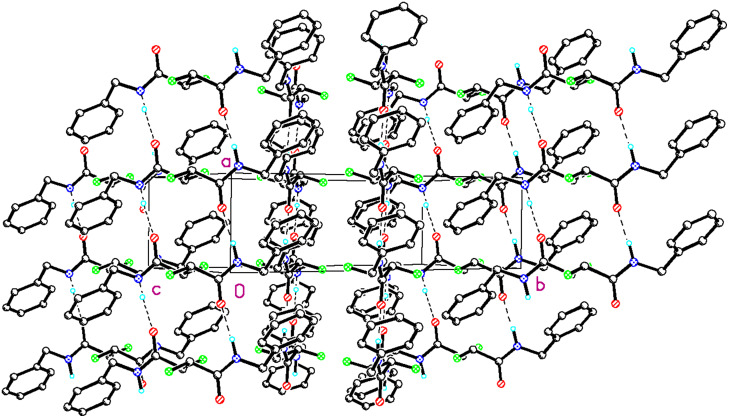
Crystal packing of *erythro*-**22** clearly indicating intermolecular hydrogen bonding.

*Erythro*- **22** adopts an extended conformation of the main chain in which the C-F bonds are *anti* with respect to each other. In that conformation the large benzyl substituents point in opposite directions. The α-fluoroamide groups tend towards a *syn*-planar C-F...N-H conformation as it is typical for this functional group (Figure 10) with the C-F bonds only 23° off the plane. The carbonyls point in opposite directions and thus intramolecular hydrogen bonding is not possible. There is however strong intermolecular hydrogen bonding between the amide hydrogen and the carbonyl oxygen of adjacent molecules which is dominating the unit cell structure (Figure 12).

**Figure 13:**
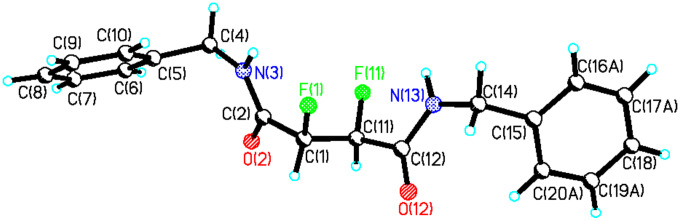
X-ray structure of *threo*-**22**.

These intermolecular interactions apparently over-ride the stereoelectronic preference for the *gauche* arrangement of the C-F bonds, which is observed in solution (*vide infra*). So we conclude that the solid and solution state structures of *erythro*- **22** are quite different. By comparison with *erythro*-**22**, the crystal structure of *threo*- **22** in Figure 13 shows both C-F bonds perfectly *syn* planar with respect to the amide N-H bonds, consistent with the typical planar arrangement of the α-fluoroamide group (Figure 10). The vicinal fluorines are *gauche* to each other. In this case the solution and solid state structures appear to be much more similar.

**Figure 14:**
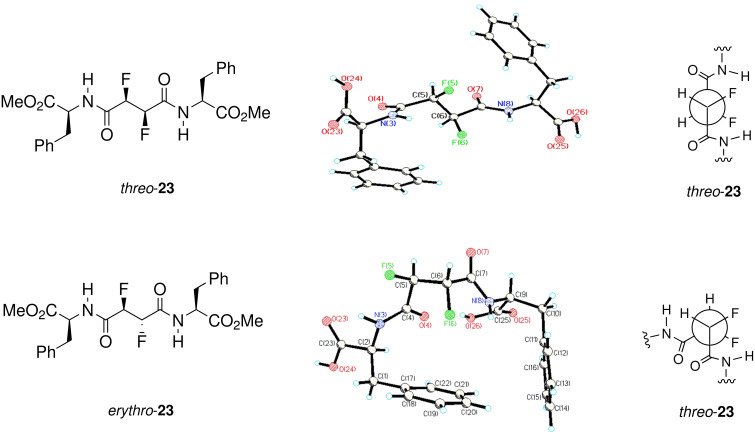
The conformations of *erythro*- and *threo*- **23** are very different as a consequence of each conformation preserving a vicinal fluorine *gauche* relationship [18].

In a recent *Communication* [18] we have reported the synthesis of peptides with 2,3-difluorosuccinic acid cores and revealed that such compounds adopt different conformations as a consequence of either the *erythro* or *threo* vicinal fluorine stereochemistry. That study highlighted both the solution and solid state conformations of the *erythro* and *threo* diastereoisomers of the *bis*-(*S*)-phenylalanine amides **23** as shown in Figure 14. The solution and solid state structures reinforced each other and the two diastereoisomers of **23** had preferred conformations where the fluorine atoms were again *gauche* to each other. This however gave very different shapes to the backbone connectivity in each diastereoisomer as illustrated in Figure 14.

### NMR studies of vicinal difluoro diastereoisomers

**Figure 15:**
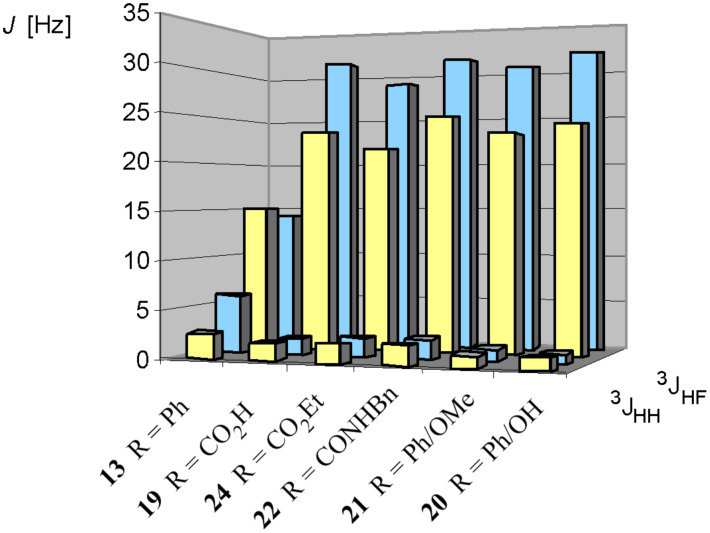
^3^*J*_HF_ and ^3^*J*_HH_ coupling constants for the *erythro* (yellow) and *threo* (blue) diastereoisomers of the 2,3-difluorosuccinates **19–22, 24** as well as 1,2-difluoro-1,2-diphenylethanes **13**. NMR spectra were recorded in CDCl_3_ with the exception of 2,3-difluorosuccinic acid, which was measured in CD_3_CN. The coupling constants were determined from second order spectra.

The vicinal difluorosuccinates again give rise to second order NMR spectra due to the chemical equivalence but magnetic non equivalence of the fluorine and CHF methine hydrogen atoms similar to Figure 3. A comparison of the ^3^*J*_HH_ and ^3^*J*_HF_ coupling constants is outlined for the vicinal difluorosuccinate diastereoisomers **19 – 22**, **24** and the 1,2-difluoro-1,2-diphenylethanes **13** in Figure 15.

Interestingly, the ^3^*J*_HF_ coupling constants are very similar to each other within each diastereoisomeric series and are essentially independent of the nature of the substituents attached to the carboxylate group. The only significant exception are the diastereoisomers of 1,2-difluoro-1,2-diphenylethanes **13** which have already been discussed in detail. For the 2,3-difluorosuccinate derivatives **19–22,24** the *threo* stereoisomers have larger ^3^*J*_HF_ coupling constants than the *erythro* stereoisomers.

**Figure 16:**
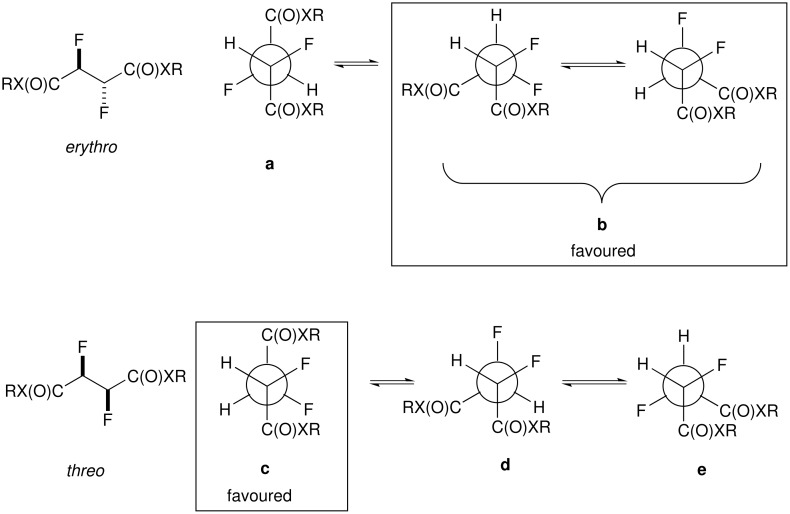
Newman projections of the three staggered conformations of the *erythro* and *threo* stereoisomers of the vicinaldifluoro compounds succinates.

In order to interpret the data in Figure 15, it is again useful to consider the staggered conformations of each *threo* and *erythro* diastereoisomer as shown in Figure 16. Each rotational isomer has two ^3^*J*_HF_ and two ^3^*J*_HH_ coupling constants the overall magnitude of each being an average of the two.

**Figure 17:**
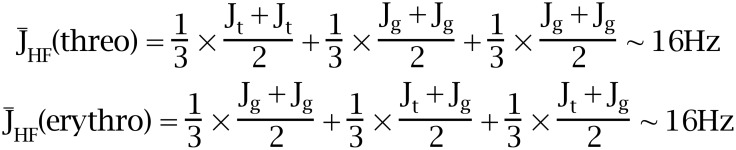
The average coupling constant with no conformational bias. The limiting coupling constants *J*_g_ = 8 Hz and *J*_t_ = 32 Hz are estimated values.

The angular dependence of the ^3^*J*_HF_ coupling constant is largely influenced by the electronegativity of the substituents adjacent to the coupling nuclei [31]. For related compounds, the full *trans* ^3^*J*_HF_ coupling constant has been estimated to be approximately 32 Hz and the *gauche* ^3^*J*_HF_ coupling constant is approximately 8 Hz [32]. With no conformational bias the average ^3^*J*_HF_ coupling constants will be (16 Hz) for each of the diastereoisomers according to these values (Figure 17).

**Figure 18:**
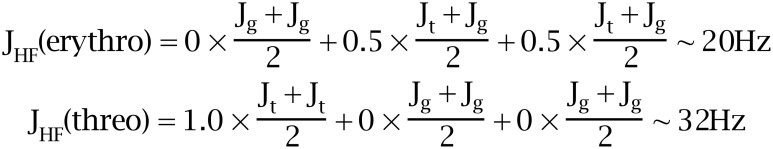
The observed ^3^*J*_HF_ coupling constants are an average over the rotational isomers.

The experimental ^3^*J*_HF_ coupling constants are clearly different for the two diastereoiomeric series. The contributions of the different conformers can then be estimated from the observed ^3^*J*_HF_ NMR coupling constants as illustrated in the equations in Figure 18.

For the *erythro* diastereoisomer the enantiotopic conformers **b** (which will be equally populated), dominate the conformer profile. This is consistent with the observed average ^3^*J*_HH_ values of (2–3 Hz) where in conformers **b** only H-H *gauche* relationships are found with no contributions from *anti* H-H couplings, which would raise this low value. The high value ^3^J_HF_ of 32 Hz for the *threo* diastereoisomers is essentially a maximum value for a *trans* coupling constant indicating the dominant contribution from conformer **c**. This is also consistent with the observed average ^3^*J*_HH_ values of (2–3 Hz) where in conformer **c** there are only H-H *gauche* relationships.

In overview the dominant conformers in each diastereoisomer series have structures which accommodate *gauche* relationships between the C-F bonds and these results suggest that the fluorine "*gauche* effect" is influencing the preferred conformations in solution. It is notable that the coupling constants for the 1,2-difluoro-1,2-diphenylethanes isomers **13** are different in the series and do not conform to the ratios described above.

## Conclusion

In this paper we have described the synthesis and comparative structures of a series of diastereoisomers of vicinal difluoro compounds, which were generated by converting stilbenes to 1,2-difluoro,1-2-diphenylethanes **13** and then oxidation of the aryl rings to generate 2,3-difluorosuccinic acids and their derivatives. The preparative methods allowed the preparation of individual *erythro* or *threo* diastereoisomers. The tendency of the vicinal fluorines to adopt predominant *gauche* conformations in solution emerges from an analysis of vicinal ^3^*J*_HH_ and ^3^*J*_HF_ coupling constants of these molecules and reinforces earlier studies on the conformation of vicinal difluoro compounds. This is in line with the well described fluorine *gauche effect*. The only exception to this was found for the *threo* stereoisomer of 1,2-difluoro-1,2-diphenylethanes **13**, where all of the data (*ab initio*, NMR and X-ray) did not converge on a consensus structure. It emerges from this study that the stereoselective incorporation of vicinal fluorines can be used to influence the conformation of organic molecules. This is an attractive tool for the design of performance molecules in areas as diverse as pharmaceutical and medicinal chemistry research to materials science.

Experimental details for the preparation and characterisation of compounds **13, 14, 15, 19, 21, 22** and **24** are given in Supporting Information File 2.

## Supporting Information

File 1 Experimental and characterisation details of synthesised compounds.

File 2 Calculated confirmations and energies.
