# Supplementary material for: The vicinal difluoro motif: The synthesis and conformation of erythro- and threo- diastereoisomers of 1,2-difluorodiphenylethanes, 2,3-difluorosuccinic acids and their derivatives
Source: Beilstein J Org Chem. 2006 Oct 2;2:19. doi: 10.1186/1860-5397-2-19 (PMC1636058; doi:10.1186/1860-5397-2-19)
Supplement: File 1 — Experimental and characterisation details of synthesised compounds. [file Beilstein_J_Org_Chem-02-19-s001.rtf]

Additional File 1
Experimental methods 

The vicinal difluoro motif.  The synthesis and conformation of erythro- and threo- diastereoisomers of 1,2-difluorodiphenylethanes, 2,3-difluorosuccinic acids and their derivatives.

David O'Hagana*, Henry H. Rzepab, Martin Schülera and Alexandra M. Z. Slawina.

a School of Chemistry, University of St Andrews, Centre for Biomolecular Sciences, North Haugh, St Andrews, Fife, KY16 9ST, UK. Fax: +44(0)1334 463808 E-mail: do1@st-andrews.ac.uk.
b Department of Chemistry, Imperial College of Science Technology and Medicine, Imperial College London, SW7 2AZ, UK.


General 
1H- and 13C-NMR spectra were recorded on either a Varian Gemini 300 MHz spectrometer (1H at 299.98 MHz, 13C at 75.43 MHz) or on a Bruker AV-300 (1H at 299.98 MHz, 13C at 75.45 MHz, 19F at 282.40 MHz) instrument. High-resolution mass spectra were obtained from Micromass GCT and LCT spectrometers. GC-MS analyses were performed on an Agilent 6890 gas chromatograph (equipped with a HP-5MS 5% PhMe Siloxane column, 1.5 µm thickness, 15m, 0.53 diameter) connected to an Agilent 5973N mass-selective detector. Infrared spectra were recorded with a Perkin Elmer 2000 FT-IR instrument using NaCl glass plates. A GallenKamp GRIFFIN MPA350.BM2.5 melting point apparatus was used to determine melting points, which are uncorrected. Crystal structure determination was performed on a Bruker SMART diffractometer with graphite monochromated Mo-Ka radiation (l = 0.7107 A) using 0.3° with steps accumulating area detector frames spanning a hemisphere of reciprocal space for both structures; the reflections were corrected for Lorentz and polarisation effects. Absorption effects were corrected on the basis of multiple equivalent reflections. 

Reaction progress was monitored on thin-layer chromatography using glass plates coated with silica gel 60 (Merck F254). TLC plates were examined under UV light (254 nm, 366 nm) and/or colouring with cerium-(IV)-sulfate stain. Column chromatography was performed on Merck silica gel 60 (60-200µm, 70-230 mesh). Eluent volumes are given as a ratio of v/v. Air- and moisture sensitive reactions were carried out under a positive pressure of nitrogen in oven-dried glassware (200 °C). All reagents are of synthetic grade and were used without further purification. Solvents were dried according to standard methods prior to use.1

Ab initio calculations
Calculations were performed using Gaussian03 (Revision C.02) using the B3LYP density functional procedure, and the two built-in basis sets: 6-31G(d,p) and cc-pVTZ. Corrections for zero point energy and entropy were via calculation of the second-derivative matrix and vibrational analysis. Solvation corrections were at the Self-consistent-reaction field (SCRF/CPCM) level for chloroform or water as solvent.


Preparation of erythro 1,2-diphenyl-1-bromo-2-fluoroethane 14
NBS (0.9g, 5 mmol) was added to a mixture of trans-stilbene (0.9 g, 5 mmol) in diethyl ether (5 cm3) and HF/pyridine (5 cm3) at room temperature. The suspension was stirred overnight and then water (10 cm3) was added. The precipitate was filtered, washed with water and recrystallised in methanol to give the title compound as a white solid (1.1g, 79%), mp 98°C (lit., 2 105 oC); (Found: C, 60.28; H, 4.21. C14H12BrF requires: C, 60.24; H, 4.33%); nmax /cm-1 (nujol) 3064, 3033, 2959, 2360, 2339, 1950, 1882, 1496, 1455 and 961; dH (CDCl3) 7.3-7.0 (10 H, m, Ar-H) and 5.8 (2H, dd, J 46.0 Hz, J 6.7 Hz, CHF) and 5.1 (2H, dd, J 15.0 Hz, J 6.7 Hz, CHBr); dC (CDCl3) 137.4, 136.3, 129.6, 129.2, 128.9, 128.7, 127.3, 127.2 (Ar-C), 95.9 (d, J 181.3 Hz, CHF) and 55.0 (d, J 28.2 Hz, CHBr); dF (CDCl3) –170.0 (dd, J 46.0 Hz, J 15.0 Hz); m/z (EI) 280, 278 (1, M+), 199 (100, M-Br-), 171 (33), 169 (32) and 109 (85). The threo-isomer of 1,2-diphenyl-1-bromo-2-fluoroethane was present as a minor component in a 1:10 ratio, dH (CDCl3) 7.3-7.0 (10H, m, Ar-H), 5.8 (2H, dd, J 45.7 Hz, J 7.7 Hz, CHF) and 5.6 (2H, dd, J 12.9 Hz, J 7.7 Hz, CHBr); dF (CDCl3) –169.5 (dd, J 45.7 Hz, J 12.9 Hz).


Preparation of E-fluorostilbene 15

Potassium tert-butoxide (0.17 g, 1.5 mmol) was added to a solution of the fluorobromide 14 (0.28 g, 1.0 mmol) in pentane (10 cm3) and the reaction mixture was heated under reflux for 5 h. The reaction mixture was then diluted with hexane (10 cm3) and water (10 cm3) was added. The organic layer was washed with brine (10 cm3) and dried over MgSO4. The solution was concentrated en vacuo to give E-15 (0.16 g, 82%) as a white crystalline solid, mp 93-97 oC (lit.,3 94-95 oC); (Found: M+, 198.0845. C14H11F requires MH+, 198.0840 ppm); nmax /cm-1 (film) 3084, 3058, 3027, 1662, 1602, 1498, 1446, 1360, 1219, 1180, 1083, 1052, 1026, 918, 867, 842, 772, 731 and 694; dH  (CDCl3) 7.5-7.1 (10H, m, Ar-H), 6.4 (1H, d, J 21.8 Hz, CH); dF  (CDCl3) –96.5 (d, J 21.8 Hz, CHF); dC  (CDCl3) 132.0-127.5 (Ar-C), 109.7 (J 31.0 Hz), 106.7 (J 256.9 Hz); m/z (EI): 198 (100), 197 (80), 196 (61), 183 (27), 178 (15) and 170 (11).

Preparation of erythro 1,2-difluoro-1,2-diphenylethane 13


NBS (3.6 g, 20 mmol) was added to a solution of trans-stilbene (3.6 g, 20 mmol) in dry diethyl ether (20 cm3) and 70% HF/pyridine (20 cm3). The reaction mixture was stirred for 3 h and Ag(I)F (2.5 g, 20 mmol) was added. The reaction then was stirred for 12 h in the dark and was quenched into H2O (20 cm3). The mixture was extracted into diethyl ether (3 x 20 cm3). The combined organic phases were washed with NaHCO3 solution (10 cm3), H2O (10 cm3) and brine (10 cm3). The solvent was dried under Na2SO4 and the removed to give the crude product. The solid was recrystallised in methanol and then petroleum ether to give a white crystalline product (3.3 g, 76 %), mp 102–103 °C (lit.,4 100-102.5 oC); (Found: C, 77.04; H, 5.53. C14H12F2 requires: C, 77.05; H, 5.54%); vmax /cm-1 (KBr) 1618, 1508, 1498, 1457, 1243, 1208, 1001, 918, 829, 761, 699 and 598; dH (CDCl3) 7.4-7.1 (10H, m, Ar-H), 5.8-5.5 (2H, AA'XX', J 45.2 Hz, J 15.2 Hz, J –16.5 Hz, J 2.6 Hz, 2 x CHF; dC (CDCl3) 129.3, 128.7, 128.6, 127.2, 127.1 (Ar-C) and 94.7 (dd, J 181.9 Hz, J 28.7 Hz, 2 x CHF); dF (CDCl3) –187.1 (AA'XX', J 45.2 Hz, J 15.2 Hz, J -16.5 Hz); m/z (EI) 218 (15), 109 (100) and 83 (8). 

Preparation of threo 1,2-difluoro-1,2-diphenylethane 13

NBS (3.6g, 20 mmol) was added to a solution of trans-stilbene 9 (3.6 g, 20 mmol) in dry diethyl ether (20 cm3) and 70% HF/pyridine (20 cm3). The reaction mixture was stirred for 3 h and Ag(I)F (2.5g, 20 mmol) was added. The reaction mixture was stirred overnight in the dark and H2O (20 cm3) was added. The mixture was extracted into diethyl ether (3 x 20 cm3). The combined organic phases were washed with NaHCO3 solution (10 cm3), H2O (10 cm3) and brine (10 cm3). The solvent was dried under Na2SO4 and removed to give a crude product. The threo-isomer 13 was purified from the 1:4 mixture of stereoisomers by a combination of crystallisation (methanol), flash chromatography (hexane/dichloromethane 20:1), and again crystallisation (hexane), mp 94 °C (lit.,3 90-91 oC); (Found: C, 77.13; H, 6.05. C14H12F2 requires: C, 77.05; H, 5.54%); nmax /cm-1 (KBr) 3033, 2970, 1495, 1214, 1074, 1004, 849 and 699; dH (CDCl3) 7.4-7.1 (10H, m, Ar-H) and 5.8-5.5 (2H, AA'XX', J 47.2 Hz, J 14.1 Hz, J –17.3 Hz, J 6.0 Hz, 2 x CHF); dC (CDCl3) 129.4, 128.7, 127.2, 127.2, 127.1, (Ar-C) and 95.46 (dd, J 184.1 Hz, J 25.4 Hz); dF (CDCl3) –187.1 (AA'XX', J 47.2 Hz, J 14.1 Hz, J –17.3 Hz); m/z (EI) 218 (7), 109 (100) and 83 (8). 

Synthesis of erythro 2,3-difluorosuccinic acid 19

A solution of erythro-1,2-difluorobiphenylethane 13 (0.630 g, 3 mmol) in acetic acid (600 cm3) was treated with ozone over a period of 16 h at ambient temperature. 30% H2O2 (100 cm3) was then added, and the reaction mixture stirred for 12 h. Platinum black (0.02 g) was added and stirring continued for 2 h to remove excess of hydrogen peroxide. When gas evolution had ceased, the mixture was concentrated in vacuum and the solid filtered off under reduced pressure. The remaining solution (50 cm3) was diluted with H2O (25 cm3) and extracted into diethyl ether (2 x 25 cm3) to remove starting material and by-products. The aqueous phase was freeze dried to give the crude product (0.208 g, 45%) as an amorphous brown solid. The product was sublimed in a Kugelrohr apparatus (100 oC/0.1 mm) to give erythro-19 as colourless crystals, mp 174-175 °C; (Found: C, 31.46, 2.69 H. C4H4O4F2 requires: C, 31.18; 2.62 H, %); nmax /cm-1 (KBr) 2946, 1719, 1450, 1271, 1240, 1122, 1096, 857 and 744; dH  (CD3CN) 5.5 (2H, AA'XX', J 46.7 Hz, J 23.3 Hz, J -14.0 Hz, J 1.8 Hz, CHF); dC  (CD3CN) 87.6 (J 191.6 Hz, J 22.9 Hz); dF  (CD3CN) –202.0 (AA'XX', J 46.7 Hz, J 23.3 Hz, J -14.0 Hz, J 1.8 Hz); m/z (ESI-) 153.22 (M-H). 


Synthesis of threo 2,3-difluorosuccinic acid 19


Ozone was bubbled through a mixture of 1,2-difluoro1,2-biphenylethanes 13 (0.440 g, 2.1 mmol) in acetic acid (400 cm3) over a period of 16 h at ambient temperature. 30% H2O2 (100 cm3) was added and the reaction mixture stirred for 12 h. Platinum black (0.02 g) was added, and the stirring continued for 2 h to remove excess of hydrogen peroxide. When gas evolution had ceased, the mixture was concentrated in vacuum and the solid filtered off under reduced pressure. The remaining solution was diluted with H2O and extracted into diethyl ether to remove unreacted starting material and by-products. The aqueous phase was freeze dried to give the crude product (0.097 g, 30%) as a brown gum. The compound was crystallised in hot acetone/benzene to give the product as light-brown crystals; mp 203-204 °C; (Found: MH+, 153.0002. C4H3O4F2 requires: 152.9999, 1.9 ppm); nmax /cm-1 (KBr) 3526, 1986, 1707, 1448, 1346, 1290, 1233, 1143, 1073, 912, 886, 830, 764, 671, 618 and 493; dH  (CD3CN) 5.5 (2H, AA'XX', J 45.3 Hz, J 31.2 Hz, J -8.8 Hz, J 1.6 Hz, CHF); dC  (CD3CN) 87.1 (dd, J 190.7 Hz, J 21.0 Hz); dF  (CD3CN) –202.0 (AA'XX', J 45.3 Hz, J 31.2 Hz, J -8.8 Hz, J 1.6 Hz); m/z (CI+) 155.02 (M+H+). 


Preparation of methyl erythro 2,3-difluoro-3-phenylpropionate 21 


A mixture of erythro and threo 1,2-difluoro-2-phenylpropionic acid was obtained after ozonolysis from ether extraction of the crude product as described in 3.16. The combined organic extracts were dried over MgSO4 and concentrated under reduced pressure. The residue was dissolved in dry methanol and heated under reflux for 12 h in the presence of Dowex 50WX8-400 ion-exchange resin (50 mg). The resin was filtered and the filtrate concentrated en vacuo to give a crude product, which was purified over silica gel (hexane/diethyl ether 10:1) to afford the title compound as a colourless oil, (Found: M+Na+, 223.0543. C10H10O2F2Na requires 223.0547, -1.5 ppm); nmax /cm-1 (KBr) 3039, 2958, 2927, 2854, 1770, 1748, 1455, 1440, 1363, 1300, 1288, 1270, 1220, 1123, 1037, 1015, 718, 699 and 558; dH  (CDCl3) 7.4-7.2 (5 H, m, Ar-H), 6.0-5.7 (1H, ddd, J 44.0 Hz, J 20.7 Hz, J 3.6 Hz, Ph-CHF), 5.2-4.9 (1H, ddd, J 49.2 Hz, J 12.5 Hz, J 3.6 Hz, CO-CHF) and 3.7 (3H, s, OCH3); dC  (CDCl3) 129.9, 129.0, 127.0, 126.9 (Ar-C), 92.2 (dd, J 180.8 Hz, J 21.6 Hz, CHF), 89.9 (dd, J 195.2 Hz, J 27.9 Hz, CHF) and 53.1 (s, CH3O); dF  (CDCl3) –187.6 (ddd, J -15.4 Hz,  CHF), –203.1 (ddd, J 15.4 Hz,  CHF); m/z (EI): 200 (M+, 2), 180 (35), 149 (21) and 109 (100).


Preparation of methyl threo 2,3-difluoro-3-phenylpropionate 21 


The title compound was isolated from the mixture of erythro and threo diastereoisomers after separation over silica gel (hexane/diethyl ether 10:1), mp 48-49 oC (Found: M+Na+, 223.0548. C10H10O2F2Na requires 223.0547, 0.1 ppm); nmax /cm-1 (KBr) 3040, 2955, 2851, 1769, 1745, 1494, 1455, 1437, 1360, 1294, 1222, 1127, 1081, 1028, 766, 714,633 and 577; dH  (CDCl3) 7.4-7.3 (5 H, m, Ar-H), 6.0-5.7 (1H, ddd, J 44.6 Hz, J 23.6 Hz, J 2.8 Hz, Ph-CHF), 5.5-5.2 (1H, ddd, J 46.9 Hz, J 26.1 Hz, J 2.8 Hz, CO-CHF) and 3.8 (3 H, s, OCH3); dC  (CDCl3) 129.8, 129.1, 129.0, 126.7, 126.6 (Ar-C), 92.3 (dd, J 182.4 Hz, J 19.4 Hz, CHF), 90.2 (dd, J 197.0 Hz, J  23.5 Hz, CHF) and 53.2 (s, CH3O); dF  (CDCl3) –192.7 (ddd, J -10.1 Hz,  CHF), –206.3 (ddd, J 10.1 Hz,  CHF); m/z (EI): 200 (M+, 2), 180 (30), 149 (22) and 109 (100).

Preparation of diethyl erythro 2,3-difluorosuccinate 24 
Erythro 2,3-difluorosuccinic acid (0.208 g, 1.0 mmol) was heated under reflux in dry ethanol (20 cm3) in the presence of Dowex 50WX8-400 ion-exchange resin (0.1 g) for 24 h. The resin was filtered off and excess solvent removed by evaporation. The residue was purified over silica gel (hexane/Et2O 10:1) to give erythro 24 (0.14 g, 91%) as a clear colourless liquid, (Found: MH+, 211.0792. C8H13O4F2 requires MH, 211.0782 ppm); nmax /cm-1 (film) 2986, 2946, 2908, 1765, 1746, 1462, 1376, 1299, 1217, 1124, 1094, 1026, 856 and 679; dH  (CDCl3) 5.30  (2H, AA'XX', J 48.0 Hz, J 21.5 Hz, J -14.0 Hz, J 1.9 Hz, 2 x CHF), 4.2 (4H, q, J 7.2 Hz, 2 x OCH2), 1.2 (6H, t, J 7.2, 2 x OCH2CH3); dC  (CDCl3) 88.3 (dd, J 196.8 Hz, J 23.2 Hz, CHF), 63.0 (OCH2), 14.4 OCH2CH3; dF  (CDCl3) –202.0 (AA'XX', J 48.0 Hz, J 21.5 Hz, J -14.0 Hz, J 1.9 Hz, 2 x CHF); m/z (EI): 210 (1), 183 (17), 165 (29), 137 (71), 109 (17), 90 (50), 73 (30) and 45 (25).

Preparation of N,N'-dibenzyl erythro 2,3-difluorosuccinamide 22

Benzylamine (0.43 g, 4.0 mmol) and HOBt (0.54 g, 4.0 mmol) were added to a solution of 2,3-difluorosuccinic acids 19 (0.31 g, 2.0 mmol) in DMF (10 cm3) at ambient temperature. The reaction mixture was cooled to 0 oC, and a solution of EDC (0.84 g, 4.2 mmol) in CHCl3 was added slowly. The reaction mixture was stirred for 24 h at ambient temperature. The mixture was then quenched with H2O (10 cm3) and extracted into ethyl acetate (3 x 10 cm3). The combined organic extracts were washed with 1N HCl (2 x 10 cm3), saturated sodium bicarbonate solution (10 cm3), and brine (10 cm3). The solvent was dried over MgSO4 and removed under reduced pressure to give the product (0.155 g, 93%) as a mixture of stereoisomers. Separation by flash chromatography yielded the erythro isomer 22 as a white solid, mp 159-160 oC (Found: C, 64.85; H, 5.32; N, 8.42. C18H18F2N2O2 requires: C, 65.05; H, 5.46; 8.43 N%); nmax /cm-1 (KBr) 3287, 3101, 3070, 3034, 2933, 2434, 1662, 1555, 1455, 1068, 1045, 1003, 747 and 695; dH  (CDCl3) 7.4-7.2 (10 H, m, Ar-H), 6.6 (2 H, m, NH), 5.4 (2 H, AA'XX', J 48.4 Hz, J 24.8 Hz, J -11.9 Hz, J 2.1 Hz, 2 x CHF) and 4.5-4.3 (4H, m, Ph-CH2); dC  (CDCl3) 137.5, 129.2, 128.2, 128.1 (Ar-C) and 91.2 Hz (J 197.4 Hz, J 22.7 Hz, 2 x CHF); dF  (CDCl3) –199.8 (2 F, AA'XX', J 48.4 Hz, J 24.8 Hz, J -11.9 Hz, J 2.1 Hz, CHF); m/z (ESI-) 331.34 (M-H+). 


Preparation of N,N'-dibenzyl threo-2,3-difluorosuccinamide 22

Benzylamine (0.43 g, 4.0 mmol) and HOBt (0.54 g, 4.0 mmol) were added to a solution of 2,3-difluorosuccinic acid (0.31 g, 2.0 mmol) in DMF (10 cm3) at ambient temperature. The reaction mixture was cooled to 0 oC, and a solution of EDC (0.84g, 4.2 mmol) in CHCl3 was added slowly. The reaction mixture was stirred for 24 h at room temperature. H2O (10 cm3) was added and the mixture extracted into ethyl acetate (3 x 10 cm3). The combined organic extracts were washed with 1N HCl (2 x 10 cm3), saturated sodium bicarbonate solution (10 cm3), and brine (10 cm3). The solvent was dried over MgSO4 and removed under reduced pressure to give the product (0.155 g, 93%) as a mixture of stereoisomers. Separation by flash chromatography yielded the threo isomer of 22 as an amorphous white solid, mp 133-147oC; (Found: C, 65.00; H, 5.50; N, 8.41. C18H18F2N2O2 requires: C, 65.05; H, 5.46; 8.43 N%); nmax /cm-1 (KBr) 3324, 1666, 1554, 1495, 1455, 1430, 1363, 1295, 1247, 1116, 1086, 1073, 1048, 1028, 862, 825, 737, 700, 620 and 575; dH  (CDCl3) 7.4-7.2 (10 H, m, Ar-H), 6.7 (2 H, m, NH), 5.3 (2H, AA'XX', J 46.0 Hz, J 31.3 Hz, J -12.6 Hz, J 2.0 Hz, 2 x CHF), 4.5-4.3 (4H, m, Ph-CH2); dC  (CDCl3) 137.4, 129.2, 128.2, 128.1 (Ar-C) and 90.0 (dd, J 198.5 Hz, J 21.0 Hz, 2 x CHF); dF  (CDCl3) –207.1 (AA'XX', J 46.0 Hz, J 31.3 Hz, J -12.6 Hz, J 2.0 Hz, CHF).

1	Perrin DD, Armarego WLF: Purification of Laboratory Chemicals, Pergamon, New York, 1999.
2	Brand M, Rozen S: J. Fluorine Chem., 1982, 20, 419 - 424.
3	Barton DHR: J. Chem. Soc., Perkin Trans. 1, 1974, 739 - 742.
4	Lermontov SA, Sergei A, Zavorin SI, Bakhtin IV, Pushin AN, Zefirov NS, Stang PJ: J. Fluorine Chem., 1998, 87, 75 - 83.
