# Supplementary material for: The vicinal difluoro motif: The synthesis and conformation of erythro- and threo- diastereoisomers of 1,2-difluorodiphenylethanes, 2,3-difluorosuccinic acids and their derivatives
Source: Beilstein J Org Chem. 2006 Oct 2;2:19. doi: 10.1186/1860-5397-2-19 (PMC1636058; doi:10.1186/1860-5397-2-19)
Supplement: File 2 — Calculated confirmations and energies. [file Beilstein_J_Org_Chem-02-19-s002.zip › ohagan/index.html]

The vicinal difluoro motif: The synthesis and
conformation of erythro- and threo- diastereoisomers of
1,2-difluorodiphenylethanes, 2,3-difluorosuccinic acids and
their derivatives


# The vicinal difluoro motif: The synthesis and conformation of erythro- and threo- diastereoisomers of 1,2-difluorodiphenylethanes, 2,3-difluorosuccinic acids and their derivatives

## David O'Hagan, Henry S Rzepa, Martin Schüler and Alexandra MZ Slawin

Beilstein Journal of Organic Chemistry 2006, 2:19
doi:10.1186/1860-5397-2-19

---

## Supporting Information

Molecular coordinates for various calculated conformations of 1,2-difluorodiphenylethane

| (R,R) Diastereomer | | | (R,S) Diastereomer | | |
| --- | --- | --- | --- | --- | --- |
| Total, Hartree,  (B3LYP/cc-pVTZ | ΔG (ZPE+Entropy),  B3LYP/cc-pVTZ | SCRF(CPCM/chloroform)  B3LYP/cc-pVTZ//cc-pV5Z | Total, Hartree,  ( B3LYP/cc-pVTZ | ΔG (ZPE+Entropy),  B3LYP/cc-pVTZ | SCRF(CPCM/chloroform),  B3LYP/cc-pVTZ//cc-pV5Z |
| **a**:  -740.66379 | -740.483483 | {3.1} -740.743031 | d: -740.664449 | -740.484945 | {0.0} -740.742732 |
|  | | |  | | |
| **c**:  -740.66382 | -740.482743 | {1.0} -740.740935 | e: -740.664119 | -740.483260 | {3.5} -740.742320 |
|  | | |  | | |
| **b**:  -740.66398 | -740.48349 | {4.5} -740.743199 |
|  | | |
